# Making Sense of the Tangle: Insights into Chromatin Folding and Gene Regulation

**DOI:** 10.3390/genes7100071

**Published:** 2016-09-23

**Authors:** Ill-Min Chung, Sarada Ketharnathan, Seung-Hyun Kim, Muthu Thiruvengadam, Mari Kavitha Rani, Govindasamy Rajakumar

**Affiliations:** 1 Department of Applied Bioscience, College of Life and Environmental Science, Konkuk University, Seoul 143 701, Korea; imcim@konkuk.ac.kr (I.-M.C.); kshkim@konkuk.ac.kr (S.-H.K.); thiruv30@yahoo.com (M.T.); 2 Department of Pathology, University of Otago, Dunedin 9016, New Zealand; sarada.biotech@gmail.com; 3 Department of Biochemistry, Periyar University, Salem 636011, Tamil Nadu, India; kavibio91@gmail.com

**Keywords:** chromatin folding, replication domains, topologically associating domains, gene regulation

## Abstract

Proximity ligation assays such as circularized chromosome conformation capture and high-throughput chromosome capture assays have shed light on the structural organization of the interphase genome. Functional topologically associating domains (TADs) that constitute the building blocks of genomic organization are disrupted and reconstructed during the cell cycle. Epigenetic memory, as well as the sequence of chromosomes, regulate TAD reconstitution. Sub-TAD domains that are invariant across cell types have been identified, and contacts between these domains, rather than looping, are speculated to drive chromatin folding. Replication domains are established simultaneously with TADs during the cell cycle and the two correlate well in terms of characteristic features, such as lamin association and histone modifications. CCCTC-binding factor (CTCF) and cohesin cooperate across different cell types to regulate genes and genome organization. CTCF elements that demarcate TAD boundaries are commonly disrupted in cancer and promote oncogene activation. Chromatin looping facilitates interactions between distant promoters and enhancers, and the resulting enhanceosome complex promotes gene expression. Deciphering the chromatin tangle requires comprehensive integrative analyses of DNA- and protein-dependent factors that regulate genomic organization.

## 1. Introduction

Genomic DNA in each human diploid cell, which is 2 m in total length, is folded around histone proteins to form the compact nucleosome, which in addition to DNA and the histone octamer core, contains the tail regions of histone proteins. The predominant histone octamer core contains two of each of the four histones, H2A, H2B, H3, and H4, around which ≈147 bp of DNA is wound. The nucleosome also contains the histone H1, which binds outside the nucleosome “bead” to the linker DNA (20–80 bp) that connects adjacent nucleosomes. In the last decade, the emergence of new techniques, such as proximity ligation assays, have provided novel and fundamental insights into chromatin organization at different stages of the cell cycle. The mechanisms by which regulatory elements such as enhancers and CCCTC-binding factor (CTCF) sites control genome organization have also been elucidated. This review focuses on recent insights into genome organization, regulatory mechanisms of genome organization, and the significance of genome organization to gene regulation, development, and disease.

## 2. Chromosome Compartmentalization in the Nucleus

**Figure 1:**
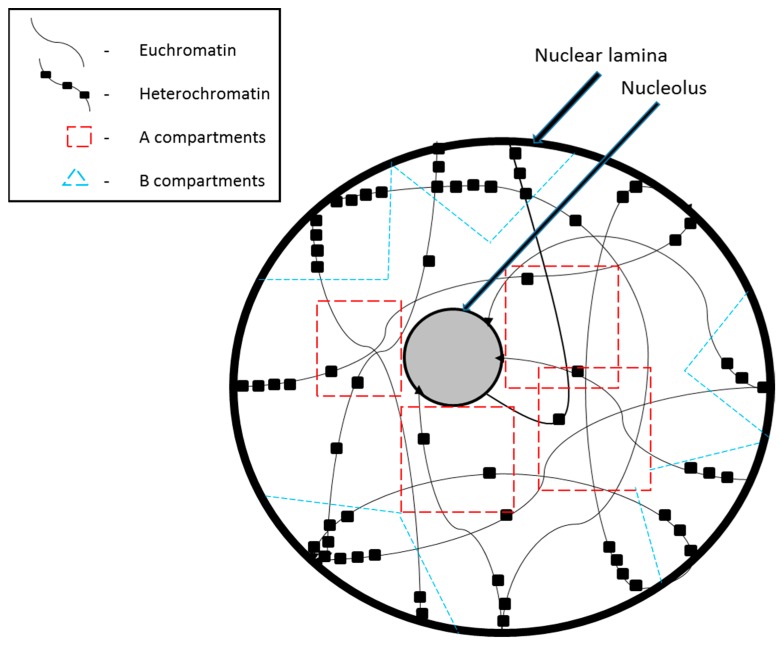
Chromatin organization in the nucleus during interphase.

The tangled mass of chromatin is spatially organized in the nucleus within very distinct topological domains. Larger chromosomes and chromosomes with more heterochromatin regions are seen to be present on the nuclear periphery while smaller chromosomes and those with more euchromatin regions are localized at the center of the nucleus [1,2]. The existence of such topological domains has been brought to light by recently developed molecular and genomic techniques such as the circularized chromosome conformation capture assays [3,4]. Based on Hi-C data, two major chromatin compartments have been defined: the A compartment that is made up of the more active and open chromatin, and the B compartment that is made up of inactive and closed chromatin (Figure 1). These compartments can be as large as several Mb; their existence was first suggested by electron microscopy analysis [5], which showed densely-stained chromatin near the nuclear periphery and nucleoli. Active chromatin is concentrated near the center of the nucleus because this is also where transcription factors (TF), RNA polymerases, and other transcriptional regulatory factors are concentrated [6]. In contrast, peripheral nuclear lamina is predominantly composed of intermediate filaments and proteins that provide mechanical support. Laminin polypeptides of the nuclear lamina have a high affinity for inactive chromatin regions with high A/T content, which are referred to as matrix attachment regions (MARs).

Within each of the A and B compartments, there are sub-domains called topologically associating domains (TADs) where interactions are at their highest levels [7]. TADs are highly conserved across various cell lines indicating that they must be important in laying the foundation for the building of the bigger compartments. The A and B compartments appear to be cell-type specific [8]. They can be reorganized to lie closer to the nuclear periphery or the center based on chromatin modifications, which in turn depend on what genes are necessary at eachpoint during development [9]. However, the extent of conservation at TADs is only valid during the interphase. During mitosis, specifically at the metaphase stage, the chromatin takes up an altogether different conformation and to achieve this, even the TADs need to be broken down. Several models have been proposed to explain chromosomal reorganization during mitosis. Electron microscopy and immunofluorescence assays suggest that chromosomes may be organized as hierarchically folded chromatin fibers or chromatin loops, held together along a central axis made of only condensin [10] or of condensin and topoisomerase II [11,12]. Through polymer simulations and genomic analysis, it was discovered that TADs are formed by progressive loop extrusion mediated by loop extruding factors [13].

Genome organization is partly driven by the high affinity of active chromatin regions for other active regions, which also explains why interactions between euchromatin and heterochromatin are infrequently observed. The interactions listed by their order of strength from strongest to weakest are: within A compartments > within B compartments > between A and B compartments [8]. Approximately 70% of chromatin looping events, which facilitate gene regulation by distant regulatory elements [14,15,16], occur within TADs [17]. The typical length scale of chromatin looping is 10–200 kb, whereas that of TADs can be as large as 2000 kb. Recent insights have suggested that TADs can be regarded as looped domains that are held together and demarcated by convergent CTCF sites. In addition, distinct domains within TADs, or sub-TADs, have been reported, which vary between cell types, unlike TADs. Gene regulation at a distance can occur through contacts between sub-TADs without requiring direct chromatin looping of gene promoters with regulatory elements; thus, chromatin folding may be based on sub-TAD interactions rather than chromatin looping mechanisms [18]. However, further studies are required to better understand thesub-TADs and their potential importance indefining genome architecture and the genomic regulatory landscape.

## 3. Plasticity of TADs

Within a TAD, the activity of individual DNA sequence elements is correlated [19], which indicates non-local TAD-specific regulatory mechanisms. Gene expression from promoter sequences in the same TAD was better correlated than gene expression from the same promoter sequences, but in different TADs [20]. TAD boundaries are typically defined by CTCF sites, promoters, and short interspersed nuclear elements (SINE), and sub-TAD regions are demarcated by CTCF and cohesin-binding sites [21]. CTCF, cohesin, and mediator proteins are critical for chromatin folding and for facilitating long-range regulatory interactions. Knockout of CTCF results in disruption of the associated chromatin loops and the dysregulation of relevant genes [22]. In addition, the deletion of CTCF sites alters the frequency of interactions between loci in adjacent TADs and hence the expression of neighboring genes [23,24,25,26]. The binding of CTCF and other specific factors may by itself intrinsically demarcate boundaries, which is likely the case at most boundaries. Boundaries may also be established indirectly by the aggregation of genetic loci present within TADs, driven by factors such as sequence homology [27].

Several inversions and deletions have been described that disrupt or modify TAD boundaries. For instance, a 0.3 Mb inversion at the *Tfap2c* (Transcription factor AP-2 gamma) and *Bmp7* (Bone morphogenetic protein 7) locus in mouse embryos results in the shifting of a TAD boundary, activation of *Tfap2c*, and silencing of *Bmp7* [28]. The loss or modification of TAD boundaries also results in the activation of oncogenes in cancer cells. TAD boundaries and specifically CTCF binding domains are frequent targets of alteration in cancer cells. In acute myeloid leukemia, as a result of chromosome inversion, two TAD boundaries are disrupted, and an enhancer is dislocated to the TADcontaining the *Evi1*oncogene [29]. However, this is not the case in the majority of other diseases, wherein single nucleotide polymorphisms are frequently found in regulatory elements rather than in TAD boundaries [30].

## 4. Overlap of Topologically Associating Domains and Replication Domains

Interphase TADs are disrupted during mitosis, requiring reconstitution of the genome architecture after each round of mitosis. Replication domains, defined as 400–800-kb DNA regions that replicate synchronously, are established during the G_1_ phase of the cell cycle [31]. At the same time, TADs and inter-TAD boundaries are also established. Regions that replicate synchronously are spatially proximal to each other. Whereas TADs are retained during the G_2_ phase, the determinants of replication are lost, which suggests that other factors in addition to the chromatin organization determine the establishment of replication domains. However, during the G_0_ phase, both TADs and replication domains are maintained.

The intimate relationship between TADs, replication domains, and transcriptional regulation is also evident in the spatial organization of the replication domains. Approximately 50% of the genome replicates synchronously (i.e., ‘constitutive replication domains’) [32]. Constitutive replication domains, which are generally transcriptionally active, are positioned near the center of the nucleus where transcriptional regulatory elements are also concentrated. The remainder of the genome replicates more asynchronously, exhibits unique compartmentalization, and is reorganized when required during development (i.e., ‘developmental replication domains’) [33]. Developmental replication domains are also translocated to the nuclear center when required. Compared to constitutive replication domains, developmental replication domains exhibit only a moderate enrichment in chromatin marks, which facilitates the switch from early to late replication, and vice versa [31,34]. A and B compartments of the genome can be subdivided into more specific compartments based on these different features (Table 1).

The concept that early acting replication domains correspond to the regions with high euchromatin content has been recently dispelled. In addition to constitutively acting genes, specific developmental genes also replicate early in specific cell types. Moreover, gene expression can be altered without any change in the replication timing [32,34]. Genes can be classified into three categories based on the correlation of expression with replication timing [35]: those that are expressed only during early replication, those that are expressed during early and late replication, and those that are expressed only during late replication. These results suggest that multiple distinct mechanisms regulate gene expression and replication timing.

## 5. Replication Origins and Nuclear Organization

Pre-replication complexes (pre-RCs) are recruited to specific chromatin sites where they further recruit additional components to form pre-initiation complexes [36,37]. Although the eukaryotic genome contains numerous origins of replication, only ≈20% are initiated during a somatic cell cycle [38]. The subset of the replication origins utilized by each cell differs [39], which could be important for the simultaneous coordination of DNA replication, transcription, and other nuclear events. A significant number of replication origins are located near transcription start sites, CpG islands, and regions exhibiting DNase hypersensitivity. Moreover, high GC content has been associated with replication origin activity [40]. The establishment of a specific chromatin structure and organization likely determines global DNA replication timing and transcription. However, the chromatin state is reciprocally influenced by DNA sequences that initiate replication, referred to as replicators [41,42]. Interactions with structural nuclear components, such as those between MARs and intermediate filaments, can also regulate replication initiation [43], and specific histone modifications are associated specifically with early or late replication [44] (Table 1). Differences in nucleotide base composition may drive the complicated yet intricate relationships between replication timing, histone modification, and genome organization. In-depth analysis of functional elements, transcriptional units, replication and topological domains have revealed variations in sequence GC content. The high amounts of GC observed at early replicating domains result from small-scale increases near functional elements. At late replicating domains a gradual accumulation of A/T nucleotides is facilitated by changes in the deoxyribose nucleotide triphosphate(dNTP) pool available during the late S phase [45].

## 6. Reconstitution of Topologically Associating Domains Following Mitosis

Although chromosomes are reorganized into chromatids during mitosis, the chromatin status of specific regions is retained [46], which could contribute to the high fidelity reconstitution of the A and B compartments. DNA sequence homology could also drive domain reconstitution. During interphase, centromeric and sub-centromeric regions can fuse to form chromocenters [47,48]. In addition, differences in the distribution of repetitive sequence elements between A and B compartments could contribute to their selective compartmentalization. Long interspersed nuclear elements (LINE) are enriched in heterochromatic regions (i.e., B compartment), whereas SINE are enriched in euchromatic regions (i.e., A compartment) [8].

The distribution of lamina-associated domains (LADs) could also regulate the chromosome position and shape in the nucleus. LADs tether chromatin to the nuclear lamina through important tethering proteins; lamin B receptor (LBR) and lamin A/C. The LBR and lamin A/C are both constituent proteins of the lamina that are sequentially expressed during cell differentiation. Deletion of both proteins causes dislocation of LADs from the nuclear lamina to the nuclear center [49]. Chromosomes that are attached to the lamina appear flattened compared to their unattached counterparts. Repressive chromatin modifications, which include histone H3 lysine 27 tri-methylation (H3K27me3) and histone H3 lysine 9 di-/ tri-methylation (H3K9me2/me3), of developmentally regulated LADs, also called facultative LADs, during differentiation is associated with the silencing of genes and the translocation of corresponding domains to the nuclear and nucleolar periphery. In vitro experiments have revealed that whereas chromatin modifications occur before mitosis, domain dislocation and changes in gene expression manifest only after the completion of mitosis [50,51,52], suggesting that active signaling mechanisms during mitosis suppress domain reconstitution.

## 7. Regulatory Elements in a Divided Genome

Enhancers are important *cis*-acting DNA regulatory elements that promote TF. Enhancers do not always affect the promoters that are closest to them in the linear DNA sequence, and may only be active in specific tissue types and at specific development stages. The first enhancer sequence, which was identified in an intron of the mouse immunoglobulin heavy chain gene, increased the transcription of various nuclear factors. Enhancers may potentially function by turning on transcription of a specific locus in more cells or by increasing the number of RNA molecules transcribed from a specific locus [53].

Enhancers are typically located several kb or Mb away from the gene promoters that they act on. The mechanisms of enhancer-mediated gene transcription at a distance have recently been elucidated [54,55,56]. Chromatin looping brings the transcription initiation factors of the pre-initiation complexes that are bound at the distant enhancer site close to the gene promoter site [57]. According to the enhanceosome model, TF are recruited to clustered DNA binding enhancer sites in an orderly and cooperative manner, and transcription occurs only when all factors are present. An alternative model is the billboard model, in which the binding and function of each TF are independently regulated. The enhanceosome model proposes that gene repression of nucleosome DNA can be overcome by the binding of factors with increasing avidity. Although short and highly degenerate TF recognition sequences frequently occur in the genome, less than half of these sites are actually occupied.

Members of a TF family may share a common DNA binding domain but may be recruited by other unique elements for distinct purposes. For example, the FOXM1 (forkhead box M1) transcription factor is recruited via protein–protein interactions with CHR (cell cycle homology region) elements rather than by interaction with the consensus RYAAAYA binding motif, to specifically regulate late cell cycle processes [58]. Analysis of the protein interactome of FOX (forkhead box) TF family members also revealed differences in protein–protein interactions depending on whether FOX TF members were chromatin-bound at the time point of the analysis [59]. Therefore, the roles of TFs in gene regulation can depend on their interactions with other proteins, including transcriptional co-regulators. TFs involved in maintaining the pluripotency of stem cells or specifying cell fates, such as OCT4 (octamer-binding transcription factor 4), can recognize binding sites in silent chromatin, which triggers chromatin remodeling and the further recruitment of other factors [60].

**Figure 2:**
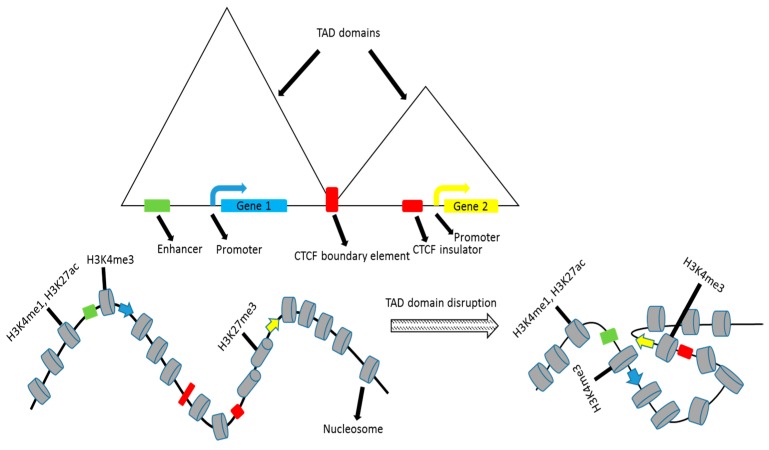
Effects of disrupting topologically associating domains (TAD) on gene regulation. CTCF: CCCTC-binding factor.

A genome-wide analysis of enhancer–promoter interactions in human primary resting CD4+ Tcells identified 2373 such interactions involving 2067 enhancers and 1619 promoters., with 91% of these being single promoter–enhancer interactions [53]. The majority of these interactions were within 50kb regions and additionally, a number of promoter–promoter and enhancer–enhancer interactions were identified. While enhancer–promoter interactions did increase gene transcription, about 23% of the enhancers were found to be bound by the insulator protein, CTCF. As CTCF is the key player in demarcating TADs, there is a good chance that the resultant increase in gene transcription is brought about by boundary disruption that brings an enhancer and a promoter in adjacent TADs closer together (Figure 2). Co-expression was seen at promoters that interact and of genes driven by a common enhancer; however, these interactions may be tissue-specific.

CTCF and cohesin act cooperatively in genome organization [61,62,63,64,65,66]. CTCF binding sites can function as insulators and block enhancer activity when inserted between a promoter and its enhancer; however, this phenomenon may be an artefact of the in vitro system it was characterized in. CTCF binding sites are concentrated in gene-dense regions and are largely tissue-invariant [67,68]. The CTCF-cohesin complex also regulates the transcription of genes in the viral genome, including those involved in pathogenicity [69,70]. CTCF sites in mammalian genomes, such as in rat, mouse, dog, and short-tailed opossum, have evolved over time through the expansion of SINE elements that carry the CTCF binding motif [71]. Epigenetic mechanisms and transposable elements also regulate genome organization and function in higher eukaryotes. In addition to its structural role in organizing sister chromatids during mitosis and meiosis, cohesin has important post-mitotic functions, which is evident from its significance in post-mitotic cells. High-affinity cohesin binding sites are associated with CTCF binding sites [64,65]. At weaker cohesin binding sites, mediator components, loading factors such as Nipbl (Nipped-B-like protein), and TF are required for efficient cohesin binding [72,73]. Thus, cohesin has CTCF-dependent and -independent gene regulatory functions [74].

Numerous databases, such as the Encyclopedia of DNA Elements (ENCODE) database, are available for predicting regulatory sequences through in silico analysis. Whereas CTCF sites are defined by a consensus sequence, enhancer regions are not. Additionally, regulatory sequences may function differently based on the tissue type and/or the developmental stage of the organism. Therefore, it will be necessary to experimentally validate computationally predicted regulatory sequence elements. Cross-disciplinary perspectives that combine structural and functional genomics will be required for a genome-wide understanding of the mechanisms of gene regulation [75].

## 8. Future Perspectives

Although proximity ligation assays have contributed to our understanding of genome organization and chromatin folding, much more remains to be discovered. Further research is required to understand how TADs reconstitute with high fidelity following every cell division. Additional studies are also required to understand the spatial and temporal relationships among replication origins, gene regulatory elements, and epigenetic modifications that regulate if and when two genomic regions are brought into close proximity. Long non-coding RNAs, which are associated with the genome architecture, may be critical to answering some of these questions. Finally, conceptual and experimental models that go beyond single-factor binding models will be required to define both the underlying molecules and mechanisms that govern genome organization.

## Figures and Tables

**Table 1:** Classification of the genomic domains based on various chromatin features.

	Replication Domains	LADs	NADs	Histone Modifications
**A1**	Constitutive early	Depleted	Depleted	H3K36me3+, H3K4me1+, H3K7me2+, H3K27ac+
**A2**	Developmental early	Depleted	Depleted	H3K36me3+, H3K4me1+, H3K7me2+, H3K27ac+, H3K9me3+
**B1**	Developmental early and late	Moderately enriched	Moderately enriched	H3K27me3+, H3K36me3−
**B2**	Constitutive late and developmental late	Enriched	Enriched	H3K36me3−, H3K4me1−, H3K7me2−, H3K27ac−
**B3**	Constitutive late and developmental late	Enriched	Depleted	H3K36me3−, H3K4me1−, H3K7me2−, H3K27ac−

LADs: nuclear lamina-associating domains; NADs: nucleolus-associating domains; H3K36me3: histone H3 lysine 36 tri-methylation; H3K4me1: histone H3 lysine 4 mono-methylation; H3K7me2: histone H3 lysine 7 di-methylation; H3K27ac: histone H3 lysine 27 acetylation.
